# Retrospect of the Medical Sciences

**Published:** 1843-07-08

**Authors:** 


### RETROSPECT OF THE MEDICAL SCIENCES.

### TREATMENT OF PNEUMONIA.

Some very interesting results have been obtained by Grisolle in reference to the influence of blood-letting on the progress of pneumonia from the first to the second stage; and as he advises blood-letting much in the same way as we do in this country, I may shortly notice his observations in order to contrast them with what he found to occur in cases treated by antimony alone. He bled forty-six cases in the first stage of the disease, and yet all, with the exception of ten passed into the second. He treated forty-four cases with antimony alone, and they were not selected because improper for blood-letting, or because the disease was trivial, for in thirty five it had already passed into the second stage, and one in seven of the whole number died. The effects of the antimony were much more strikingly beneficial than those of the blood-letting had been in the other cases. In those who recovered, the principal *symptoms* began to amend on the first or second day for the most part, and the local signs continued to increase for several days only in a single instance, while in eighteen cases they began to improve at the end of the first day. That blood-letting should have been practised in some of these cases he admits, referring more especially to three of the six cases that died, in which he says that the circulation was unusually vigorous. We learn from these various researches, not that the one or other practice should be exclusively adopted, but that it is not necessary to repeat the blood-letting so often as the patient seems capable of bearing it—that much of the cure may be safely entrusted, in a great many cases that would still admit of depletion, to the antimony—and that, since antimony alone is evidently so potent a remedy, we have no reason to despair of ultimate recovery in cases that will not bear the evacuation of blood—a remark which can seldom be made of any other considerable inflammatory disease than pneumonia.—*Prof. Henderson, Lond. and Edin. Monthly Journal.*

---

### SOMNAMBULIC DOGS.

One of the most interesting and (as we are gravely informed) convincing proofs of the truth of Mesmerism, has recently been crushed in the bud by the ignorance and perverse activity of the French police.

Driven from the scientific societies, and finding that public credulity was becoming rather slack, the Mesmerists hit on a novel expedient, which at least had the merit of silencing all accusations of trickery and collusion. They hired a small theatre, and brought out a company of somnambulic dogs. But the wickedness of man respects nothing, and the brightest plans are often blasted by the most ignoble of persecutors. Scarcely had the actors placed their four paws on the stage when the *sergens de ville* rushed in, and consigned both beasts and Christians to the safe keeping of the station-house.—*Prov. Med. Journ.*

---

### COLD INFUSION OF CINCHONA CORDIFOLIA.

Mr. Battley has given, in the "Medical Gazette," an account of a preparation called Liquor Cinchonæ Cordifoliæ, consisting of a concentrated cold infusion of that variety of Peruvian bark. The writer believes that the value of the yellow bark has been too generally and too exclusively attributed to the quinine it contains. A series of experiments has afforded the following "pharmaceutical analysis" of this species of Cinchona bark:—

A peculiar acid, tannin, aromatic principle (essential oil?), lime, potash, alumina, iron, hydrochloric and sulphuric acids, silex, wax, resin, gummy matter, starch, woody tissue.

"The most agreeable cold infusion, and that which is most likely to suit a weak stomach, is formed by half an hour's infusion. Little or nothing is gained by continuing the maceration after the end of three hours in the same fluid; if the fluid be then poured off and a fresh proportion of water added, the maceration may be repeated for the same time. The acid, quinine, tannin, and aromatic principles, are taken up rapidly from the commencement of the operation; *the lime somewhat later.* The tannin taken up by the water apparently forms subsequently a new combination, which diminishes its effects on the salts of iron; for after six hours maceration the infusion, which had previously given a copious precipitate with the muriated tincture of iron, ceased to do so, though it acquired the green colour."

The conclusions arrived at by the anthor are as follows:—

1. Bark will yield to *cold distilled water* all its constituents except starch and woody fibre, some earthly salts, and a small portion of tannin and quinine, which can only be separated from the tissue by means of an acid.

2. Twenty-eight pounds of good yellow bark will yield from five to six pounds of concentrated liquor, specific gravity 1200, containing about ten ounces of quinine; the aroma, and the greater part of the tannin and iron, and the peculiar acid of bark, of which only a small portion is lost, forming an inert salt with lime.

3. To form this liquor it is only necessary to subpulverise the bark, and macerate it from four to six hours in twice its weight of cold distilled water, repeating the process twice, or at most thrice; to concentrate the infusions over a water bath to specific gravity 1200, and allow the liquor to deposit the gummy matter and so much of the tannin as it cannot retain in solution.

To separate any gum that may still remain in the liquor, and, to prevent any decomposition, proof spirit is added to it until the specific gravity of the liquor is reduced to 1100. The quinine still remaining the bark may, if it be thought desirable, be separated by acetic acid, and precipitated from its solution by ammonia, and, being redissolved in a small quantity of dilute acetic acid, may be diffused in the liquor.

The advantages of this medicine are—

1. That it contains not one, but all the active principles of yellow bark.

2. That the great part of the quinine is preserved *in its natural state* in combination with the peculiar acid of bark, in which it is more soluble than in sulphuric acid.

3. That the active principles *have undergone no change*, either from exposure to too great heat, as in the decoction, &c., or by being brought into too close contiguity, as in the extract, in which secondary formations take place to so great an extent that water is incapable of redissolving it.

4. That, containing no starch and little gum, it will remain unaltered for a great length of time.

5. That the quantity of spirit contained in a dose is too small to be objectionable.

6. That it is a convenient, agreeable, and elegant medicine, miscible with wine or water in any proportion.

This cold infusion of Cinchona cordifolia is stated to agree with the stomach in those cases in which the exhibition of quinine would give rise to irritation and nausea, and not unfrequently succeeds in obstinate periodical and cachectic diseases, when the disulphate of quinine has failed.—*Provincial Medical Journal.*

---

### A HINT TO MAGNETISERS.

M. Richard, professor of magnetism, and Mlle. Virginie Plain, somnambulist, have been condemned to six months imprisonment by the police magistrates at Niort, (France,) under an accusation of "swindling."—*Ibid.*

---

### COMPARATIVE WEIGHT OF THE HUMAN BRAIN IN THE MALE AND FEMALE.

Dr. John Reid has published a very interesting and valuable series of tables demonstrating the average weight of the several organs of the human body. Of those applying to the developement of the brain, the first table exhibits the heaviest, lightest, and average weight of encephalon, cerebellum, and cerebrum, with pons varolii and medulla oblongata at different ages in 253 brains. Though individual female brains are not unfrequently found to be heavier than individual male brains, yet the average male brain is several ounces heavier than the average female brain. The examination of the tables do not afford any support to the supposition of some, that the cerebellum attains its maximum weight at seven years of age, and the cerebrum its maximum weight nearly at the same period, or a little later. There appears to be little doubt, however, from all the facts which have been collected on this subject, that the brain arrives at its maximum weight sooner than the other organs of the body; and to judge (says Dr. R.) from a few measurements we have made of the length of the corpus callosum, the depth of the grey matter, the length, breadth, and depth of the corpus striatum and thalamus, we would be inclined to conclude that the relative size of the parts is the same in the young person as in the adult. We believe that there can be little doubt that the relative size of the brain to the other organs and to the entire body is much greater in the child than in the adult. We find less difference between the relative weight of the encephalon and cerebellum, at different periods of life, than we had been led to expect from some statements which had been made upon this question. The data we have collected do not entitle us to speak positively, but as the other statements to which we refer seem principally to rest upon the vague and uncertain measurements of the eye, we may reasonably request to be allowed to suspend our opinion of their accuracy, until we have a sufficient amount of materials brought before us to justify us in giving a decided judgment. In females we find a decided diminution in the average weight of the brain above sixty years of age, for we are perfectly satisfied, as the tables containing the individual facts will show, that we more frequently meet with a greater quantity of serum under the arachnoid and in the lateral ventricles in old people than in those in the prime of life. We are also satisfied, from an examination of the notes we have taken at the times the brain were examined, that a certain degree of atrophy of the convolutions of the brain over the anterior lobes, marked by the greater width of the sulci, was more common in old than in young persons. We have, however, frequently remarked these appearances in the brains of people in the prime of life, who had been for some time addicted to excessive indulgence in ardent spirits.

In 87 brains, 34 of which were obtained from females, the total ages of both male and female brains ranging from twenty-five to fifty-five, the results were—

| | Male. | | Female. | |
|---|---|---|---|---|
| | oz. | dr. | oz. | dr |
| Average weight of encephalon | 50 | $3\frac{1}{2}$ | 44 | $8\frac{1}{2}$ |
| Average weight of cerebrum | 43 | $15\frac{3}{4}$ | 38 | 12 |
| Average weight of cerebellum | 5 | 4 | 4 | $12\frac{1}{4}$ |
| Average weight of cerebellum with pons varolii and medulla oblongata - - - | 6 | $3\frac{1}{4}$ | 5 | $12\frac{1}{3}$ |

Relative weight of encephalon to the cerebellum was as 1 to 9 4-7ths in the male; in the female as 1 to $9\frac{1}{4}$. Relative weight of encephalon to cerebellum with pons and medulla as 1 to 8 8-13ths in the male; in the female as 1 to 7 9-10ths. From which it would appear that in the female the average cerebellum is relative to the encephalon a little heavier than in the male. By a table, containing the relative weights of the entire body to the encephalon, the heart and liver, in the two sexes between twenty-five and fifty-five years of age, it would appear that though the average male brain is absolutely heavier than that of the female, yet that the average female brain relative to the weight of the whole body is somewhat heavier than the average male brain.—*Edin. and Lond. Monthly Journ.*

---

### PROGNOSIS OF PNEUMONIA.

Statistical investigation proves that age, sex, and the seat of the pneumonia, have a remarkable influence on the mortality. In respect to the first, it is ascertained that pneumonia proves fatal in an increasing proportion after the twentieth year, infancy being excepted; while Chomel found the mortality but 1 in 30 of persons before twenty, it rose to 1 in 8 between twenty and forty, and to 1 in 5 between forty and sixty. Grisolle also found the mortality between fifty and sixty 1 in 5. In regard to sex, it appears that females are much less favourable subjects for pneumonia than males, the mortality among them having been, in Grisolle's extensive experience, from one-third to one-half greater. Andral first expressly declared pneumonia of the summits of the lungs to be more fatal than that of other parts—a circumstance which Louis affirms to be owing more to the well known greater frequency of pneumonia of the summits in persons advanced in life than to the mere influence of the parts affected. In this, however, he is not altogether correct, since it appears from Grisolle's cases that while 1 in 8 died from pneumonia of the lower lobes of persons of the average of thirty-seven or thirty eight, 1 in 5 died from pneumonia of the summits at the same period of life.
*Prov. Med. Journ.*

---

### EXTRACT OF A LECTURE ON SYPHILIS.
BY DR. PHILLIP RICORD.

It is, gentlemen, to meet some of the difficulties previously mentioned that I have determined, upon mature reflection, to dedicate one entire lecture to the most proper mode of investigating a case of syphilis, and to demonstrate to you the degree of confidence which may be placed in the assertions of patients.

But, before I enter upon this subject, I beg distinctly to state my most unqualified respect for those convictions, without which the very existence of constituted societies is at stake. For female virtue—that guardian of the honour of men—I have the deepest admiration; but recollect, gentlemen, it is not with homilies on chastity that a course of syphilis can be carried on; and although I conceive it to be obligatory on the physician in practice not to destroy those illusions on which, occasionally, the peace of a family may depend, but to palliate the painful truths which might cause the annihilation of individuals, yet I conceive it to be my duty, in this amphitheatre, to raise the curtain, because science must not be made to consist of the delusions of love-sick youths, but of all the elements of truth which can be collected by manly and searching intellects.

Every day, gentlemen, persons affected with syphilis deny the possibility of the fact, from their knowledge of the "respectability" of the lady who has honoured them with her favours. Let us, therefore, examine what conditions can place a woman above suspicion.

Is it the fact of her being free from the sacred bonds of matrimony ? This question I shall answer with an anecdote borrowed from Dupuytren's practice. That great surgeon performed suture on a lady in whom the perineum had been torn during parturition. A couple of years afterwards a friend and patient requested the professor to visit his young wife, with whom the happy bridegroom had been unable to effect complete intercourse. Dupuytren, to his great surprise, recognised in the "too perfect" maid the lady whom he had previously attended, and found he had been himself the involuntary cause of the deluded husband's ill success; the sutures had, in fact, caused the partial obliteration of the genital organs. He remedied the injury, and, of course, was discreet.

In consequence of the brutal and stupid superstition prevalent amongst the lower orders, that connection with a "real maiden" is the only effectual way of removing inconvenient syphilitic symptoms, I have myself seen a little girl, not eighteen months old, with chancres and buboes.

A man servant called on me not long since, labouring under gonorrhœa. His first statement was, that six months had elapsed since the last sexual intercourse. On being hard pressed with questions, he admitted that, being about to be married (two banns had been already proclaimed,) he had considered himself justified in levying a tythe on his future rights. I examined the "blushing bride," and, after much difficulty, she acknowledged that violence had been lately used towards her by another person; but, added she, to a certainty he was not diseased, for if he had been, she would positively have resented his rudeness by "raising the house." I need not tell you, Gentlemen, the third bann never was published.

Two physicians, beside myself, attended a young gentleman with constitutional syphilis. He mentioned having had intercourse with two women of suspicious character, whom the medical attendants examined separately, and found healthy. A month elapsed, and I at last discovered the real cause of this gentleman's amorous misfortunes to lie with a *married* lady of unblemished repute and high station, who had sent for me, not in my medical capacity, for she did not imagine herself infected, but to secure my attentions for the young gentleman above mentioned, whose disorderly conduct gave her no small uneasiness.

Who has not heard the famous history of Sidrac, who was presented with chancres in return for his first visit to the connubial bed. "It is true," says Voltaire, "it was a family complaint."

I must say, gentlemen, it is my conviction, more syphilis has its origin beyond the pale of prostitution than within it. But, even in Paris, what degree of security can public health derive from the medical examination of prostitutes once a week, or of unlicensed women once a fortnight, when we know *three days* to be amply sufficient for a sore to be communicated, and rendered inoculable, when we know from *our own* experience that infected women send to the physician in their stead one who does not fear inspection, and who thus brings home to them a stamped and signed permission to inflict on the unguarded and the imprudent the most horrible disease with which Providence has been pleased to chastise mankind !

The "profession" of syphilitic patients is occasionally brought forward as an unanswerable proof of the spontaneous developement of the disease, or at least as sufficient evidence of contamination, without the participation of the patient. Now, notwithstanding my profound and sincere respect for religious institutions, I conceive it would be offering an insult to your better judgment, were I to propose to you seriously, as Fallopia does in a sartirical mood, in explanation of venereal symptoms observed in a community of nuns, "the promiscuous use of holy water!"

My colleague, Dr. Serres, one of the most learned and distinguished members of the Academy of Sciences, Paris, was called in to attend a young lady who was troubled with hæmorrhoids. These turned out to be *bona fide* "chancres." The Doctor could not credit the evidence of his senses; he held a private conference with the young lady's mother, and, to his utter dismay and confusion, found her infected also, but in a manner more consonant with general custom. Further investigation showed that a *reverend* adviser of this interesting family had extended his attentions to both mother and daughter, and had left after him most painful marks of his kindness.

In the investigation of patients, physicians are often exposed to infection. I will select one out of many well authenticated instances. Our late lamented colleague, Dr. Hourmann, of the Hopital de l'Ourcine, in examining a syphilitic woman, inoculated a chancre on his finger. Secondary and tertiary symptoms were the consequence, and after ten months the disease terminated fatally.

This, however, is far from being the most frequent mode in which professional persons are diseased. A midwife with a papular and scaly eruption, was lately considered by Dr. Casenave as an instance of infection by *simple absorption* from the finger; she, of course encouraged the belief. On being cross-examined by myself, she [illegible]knowledged, with tears, that one single, never-since-repeated breach of her marriage articles had been visited on her thus severely, and that she would take good care to be perfectly faithful for the future to her lord and master.

When all these circumstances are reflected upon—when it is recollected that illicit and disgusting practices, too common in highly civilized countries, often produce primary sores of the anus, mouth, &c.—that a sense of shame will compel such patients to conceal the disgraceful truth, unless the avowal be actually wrung from them by the fear of consequences—can it be wondered at if the true origin of many cases of syphilis remains shrouded in doubt?—but is that origin, therefore, to be denied?

A class of females to which we are all, more or less, indebted—nurses—often lay upon their unconscious suckling the blame which more properly rests with older offenders. I have had under my care an infected nurse, who threw the entire onus upon an innocent baby, although she had taken, at the same time, "to nurse," an unlimited number of bold dragoons. A few days since a patient left our wards, cured of constitutional symptoms; his wife had communicated to him an indurated chancre some five months before. Here, again, a child she had nursed three years previously was made the scapegoat, and the poor husband never once reflected he had been living in the dangerous vicinity of a garrisoned town.

In the examination of syphilitic patients, we must not, gentlemen, lose sight of the disease previous to the present one—of the period at which the present symptoms may have arrived—nor of the possibility of total disappearance of former accidents. You must not forget that a phymosis may conceal the primary sores; that they may lurk within the folds of the vagina, or even be engrafted on the os uteri itself. This last remark leads us to our concluding observation, that all syphilitic cases in women, where the use of the speculum has been neglected, shall be for us as though they never were recorded.—*Prov. Med. Journ.*

---

### INDICATIONS AFFORDED BY THE STATE OF THE IRIS IN CASES OF CEREBRAL LESION.

In the examination of cases of injury to the brain it is extremely important for you to enter minutely into all those signs which indicate any injury to the brain. First, the mental condition; next, the state of the pupils—the iris is placed before that expanded surface of the optic nerve, the retina, as an intelligent curtain to guard it from injury. The vital contrivances by which it acts, and by which its action is directed, are so beautifully perfect that the extent of the opening of the curtain is indicative of the state of the nervous apparatus it is destined to protect, by preventing such an amount of light impinging upon it as would be liable to injure it. In disease of the globe of the eye the dilated pupil indicates more or less pressure on the retina by some cause in the globe itself, such as a permanently turgid choroid, &c. But if with a healthy eye, but in connection with a blow on the head, we find a dilated pupil, then we have the sign of some pressure or injury to the nerve in its course within the skull, or the ganglia in which it terminates.

The dilated pupil, then, indicates very serious injury to the optic nerve, or the nervous centres with which it is connected, though it may happen, as in the case of very severe concussion, the injury is remediable. The contracted pupil, on the contrary, indicates an irritability of the nervous instruments, an undue excitement of their natural function—not an obliteration of it. You will sometimes see, in the case of injury of the brain, dilatation of one pupil and contraction of the other; where this is the case you will find the most severe injury of the brain on the side opposite the dilated pupil. I have several facts to prove this assertion, which I shall relate on a future occasion.—*Medical Gazette, Mr. Solly's Clinical Lecture.*

### ONYXIS—THE NAIL GROWN INTO THE FLESH.

M. Gerdy does not approve of the avulsion of the nail as a means of curing this painful affection. The nail soon grows again, and with its growth returns the disease. Neither does he approve of the plan of contracting the point of the nail. Matters soon become as bad as ever. Neither is the plan of proceeding, according to which the diseased cushion of parts to the outside of the nail, although superior to the other two, to be greatly commended. The continual pressure upon the pulp of the toe by and by forces a new portion of flesh into contact with the nail, and renewed pain demands renewed interference from the surgeon. "The true and rational method of dealing with the affection," says M. Gerdy, "is to remove at a stroke, and obliquely, even beyond the middle of the pulp of the toe, the lateral and inferior parts which rise to suffer pressure from the edge of the nail, and to ulcerate under it."—*Lond. and Edin. Monthly Journ. of Med. Sci.*

---

### ON THE COMPARATIVE FREQUENCY OF THE MORNING AND EVENING PULSE.

BY DR. STRATTON.

The old opinion on this subject was, that the pulse in the morning is slower than in the evening. Dr. Knox was the first who denied this; and from experiments performed by Dr. Stratton, on himself, he comes to the same conclusion. The following are the different views on this question:—

1st. The pulse is slower in the morning than in the evening.

2d. That it is of the same frequency in the morning and evening.

3d. That it is slower in the evening than in the morning.

4th. That in two-thirds of the cases it is slower in the evening, and in one-third it is quicker,

To these, Dr. Stratton is inclined to add a

5th. That out of seven days it is quicker in the morning on six, and slower on one day.

To give a more precise idea of Dr. Stratton's experiments we insert some of his tables.

Table 1st. In thirty-four daily observations, (from the 18th of November to the 24th of December, 1841,) on twenty-nine days, the morning pulse was higher than the evening, and on five days it was lower.

| | Beats. | Difference. |
|---|---|---|
| The highest morning pulse was | 91 | 4 |
| " evenin " | 87 | |
| The lowest morning " | 68 | 5 |
| " evening " | 63 | |
| The average of the morning pulse was | 82 | 9 |
| " evening " | 73 | |

According to this series, the morning pulse is higher than the evening, except once, in nearly every seven days, or in six days and four-fifths more correctly. The other tables show nearly the same result, and we shall merely give

Table 7th, which is the mean of the experiments made by Drs. Knox, Guy, and Stratton.

| | No. of Observations. | Morning Pulse. Higher. | Lower. |
|---|---|---|---|
| Dr. Knox, | 7 | 7 | 0 |
| Dr. Guy, | 3 | 2 | 1 |
| Dr. Stratton, | 7 | 6 | 1 |
| | — | — | — |
| | 17 | 15 | 2 |

So that by this table the morning pulse is higher than the evening, except once in eight and a-half, or twice in seventeen days.

It would be desirable to have additional observations on the pulse in a state of disease, and on the pulse of women, both in health and disease.—*Abridged from Edinburgh Med. and Surg. Journ.* for Jan., 1843.

RESULTS OF HOMŒOPATHY.

We regret to state that another of the Earl of Denbigh's family has fallen a victim to the theory of infinitessimal doses. His lordship's third son died, last week, from accumulation of mucous in the air passages, the result of hooping cough. An emetic is usually employed in cases of this kind to free the bronchia; but whether the homœopathic fractions of ipecacuanha produced this effect or not we have not heard.—*Prov. Med. Journ.* May 20, 1843.

ANALYSIS OF THE BLOOD IN SIX CASES OF MORBUS BRIGHTII.

In the first case the patient was a male, aged fifty-six. The blood was very slightly buffed; the serum had a specific gravity of 1023.

In 1000 parts of blood:—

| | |
|---|---|
| Water - - - - - | 853.11 |
| Solid matters of serum - | 81.28 |
| Fibrin and corpuscles - | 65.61 |

In 1000 parts of serum:—

| | |
|---|---|
| Albumen - - - | 68.5 |
| Urea - - - - | 0.5 |
| Alkaline salts - - - | 6.0 |

In the second case the patient was a male, aged thirty-two. The blood was both cupped and buffed.

In 1000 parts of blood:—

| | |
|---|---|
| Water - - - - - | 835.85 |
| Solid matters of serum - | 82.54 |
| Fibrin and corpuscles - | 81.61 |

In the third case the patient, a male, aged thirty-five, the subject also of phthisis.

In 1000 parts of the blood:—

| | |
|---|---|
| Water - - - - - | 828.92 |
| Solid matters of serum - | 76.98 |
| Fibrin and corpuscles - | 94.10 |

In the fourth case the patient, a male, aged sixteen, the blood being cupped and buffed, the specific gravity of the serum 1018, containing, in 1000 parts, albumen 64, and salts 6.5 parts; urea detected, but not estimated.

In 1000 parts of the blood:—

| | |
|---|---|
| Water - - - - - | 777.06 |
| Solid matters of serum - | 71.14 |
| Fibrin and corpuscles - | 151.80 |

In the fifth case the patient, a male, aged thirty-two, the specific gravity of the serum 6025, containing 72 parts of albumen, and 1.25 parts of urea in 1000.

| | |
|---|---|
| Water - - - - - | 782.86 |
| Solid matters of serum - | 73.14 |
| Fibrin and corpuscles - | 144.00 |

In the sixth case the patient, a female, aged thirty-seven, the specific gravity of the serum 1029 to 1030, containing albumen 79.5 parts, and alkaline salts 7.5 parts in 1000.

In 1000 parts of the blood:—

| | |
|---|---|
| Water - - - - - | 805.71 |
| Solid matters of serum - | 85.56 |
| Fibrin and corpuscles - | 108.73 |

*Dr. Rees, in Guy's Hospital Reports.*

COMPOUND FRACTURE OF LEG.

At the Academy Sciences, Paris, April 17, 1843, M. Guyon forwarded a case illustrative of the effects of cold water in very severe injury to the limb. The case was one of fracture of the middle third of the tibia, with dislocation of the lower end of the fibula, laceration of the integuments, through which the end of the bone had passed, and compound dislocation of the astragalus. The foot was turned inwards to a right angle with the leg; the articulation of the joint was opened.

The surgeon commenced by returning the astragalus to its place, a matter of considerable difficulty; the foot was then brought outwards, the fibula replaced, and the lacerated integuments united by three stitches. There was a good deal of difficulty in keeping the foot straight, because the wound on the outer side of the ankle prevented the application of any hard body over this point; the splint used by Sir A. Cooper for fracture of the lower extremity of the tibia was at last tried, and found to answer. Irrigations with cold water, containing a small quantity of acetate of lead, were now employed without interruption for twenty days; the local symptoms were insignificant, but the general disturbance severe. On the fifteenth day some symptoms of tetanus appeared, and continued for two days, but yielded to large doses of opium. On the 5th of February the patient was in a excellent state, and the wound nearly healed.—*Prov. Med. Journ.*

TREATMENT OF TINEA FAVOSA.

The mode of treatment of this obstinate disease, employed by the freres Mahon, although kept secret by them, has for a long time been followed with unquestionable benefit in the Parisian Hospitals. The records of the bureau central, for example, prove that by this method were cured amongst others, three patients who had been unsuccessfully treated by the *calotte;* eighteen children dismissed as incurable from St. Louis; nine children, also, dismissed as incurable from the *enfans malades*, &c. Numerous other proofs might be adduced in favour of this method, a description of which we here subjoin.

The hair is first cut short, and the crusts then removed by emollient poultices. The head is now frequently washed with soap and water, and the inunctions and lotions continued until the scalp is completely cleaned. When this has been effected, the second stage of the treatment commences, the object of which is to remove the hair, *slowly* and *without pain*, from all points of the scalp occupied by the favus. Every second day the ointment (No. 1) is applied, and its use continued according to the obstinacy of the case. On the intervening days the hair is combed with a fine comb, to remove the loose hairs. This mode of treatment having been continued for about a fortnight, a depilatory powder (No. 2) is sprinkled through the hair once a week; on the following day the hair is combed, and the depilatory ointment applied as before. At the end of a month or six weeks a more active ointment is employed every day; and as the disease gives way the frictions are made only once a week, until the redness of the skin has entirely disappeared.

Although the formulæ of the remedies employed by the *freres Mahon* have been kept secret, yet their composition has been very nearly ascertained by experiment, and are supposed to be as follows:

No. 1.—Slaked lime, 8 scruples,
Soda of commerce, 12 scruples,
Lard, 64 scruples.
No. 2.—Wood ashes, 64 parts,
Pulverised charcoal, 32 parts.
*Lotion.*—Lime water, 500 parts,
Sulphate of Soda, 185 parts,
Alcohol, 24 parts,
White soap, 10 parts.

*Ibid, from Duparc on Cutaneous Diseases of Children.*

---

*Observations on the Microscopic Characters and Manner of Developement of the Blood Corpuscles.* By Dr. Martin Barry.

Dr. Martin Barry, in a paper read before the Royal Society, has thus described the development of the blood corpuscle:—" 1. The mammiferous blood corpuscle, like one of the cells of the ovum, is at first a disc or what is now called a 'cytoblast'—*i. e.*, a cellgerm. It is now a flattened vesicle or cell: like other cells or cytoblasts, however, it may and does become a cell, but then it is no longer flat. In the blood disc you see a central, colorless, concave portion, around which lies the red coloring matter.

2. As usually met with the blood disc is *round*, with the exception of two or three instances, in which, from the observations of Mandl in France, and Gulliver in this country, it has been discovered to be *elliptical.* I have since found that even in mammals, where the blood disc is usually met with round, *its original form is elliptical.* I have seen this to be the original form of the blood disc in man.

3. The discs first become round, continuing flat: subsequently, they pass into an orange shape; and, lastly, become globules. They also very much increase in size.

4. Along with these alterations in the form and size of the blood discs there takes place another change: instead of a mere concavity, there is now seen a colorless, pellucid semi-fluid substance, which, as the corpuscle becomes orange shaped, is found to be, not in the centre, but *on one side.* It is the nucleus of the corpuscle, the corpuscle itself having become a cell. This pellucid substance, or nucleus, divides into and gives off globules. Each globule, appropriating to itself new matter becomes a disc; and each disc, undergoing changes like the first, gives origin to other discs, a group of which constitutes the colourless corpuscle of the blood, for with the changes now mentioned the red colouring matter is consumed.

Thus, as the red pass into the colourless corpuscles, there must exist all intermediate stages; between them no line of distinction can be drawn.*

5. The corpuscles of the blood are propagated by means of parent cells—a parent cell has its origin in a colourless corpuscle, this colourless corpuscle being an altered disc. As the parent cell is forming, the new discs within it gradually become red, and are at length liberated to give origin in like manner to new discs, or to be appropriated in some other way.

6. From observation 4 it will be seen that the disc, or so-called cytoblast, is originally a pellucid globule, which globule, therefore, is the true cell-germ.

7. Sometimes the quantity of the pellucid substance in the blood-cell is very much increased. This takes place at the expense of the red colouring matter which surrounds it. The blood corpuscles, now cells, I have seen in various parts collected until the capillaries were completely *filled* with them, and until they had become pressed together into many-sided objects.

8. The originally colourless substance derived from the nuclei of the blood cells, and nearly filling the capillaries, as I have found it, appears to constitute the essential part of coagulable lymph to organise the same, and to give origin to the tissues, &c. It seems to be this same colourless substance derived from the nuclei of the blood-cells that forms the exudation, corpuscles of authors, the fibres of false membrane, and the filaments in coagulating blood—filaments which, as I have shown, here and there arise while this substance is still within the cells.—*Ibid, from Phil. Mag.*

---

TYPHOID FEVER AMONGST DOMESTIC ANIMALS.

M. Rayer relates a case which he considers illustrative of the existence of typhoid fever amongst animals. A young ass, six weeks old, died after having laboured under diarrhœa for about eight days. On examining the carcase, M. Rayer found no other lesion than that which is observed in the bodies of persons cut off during the first stage of typhoid fever. Several patches of the glandulæ agminatæ were enlarged, and much injected, with inflammation of the surrounding mucous membrane, and one of the patches was ulcerated in its middle. The condition of the injected mucous membrane was quite different from that observed in cases of dysentery.—*Ibid.*

---

TURNING OF PLANTS TOWARDS THE LIGHT.

At the Academy of Science, Paris, May 8, 1843, M. Becquerel read a report on a memoir by M. J. Payer on this subject, The chief result obtained by the experiments of the author, verified by the committee, is that the portion of the spectrum comprised between the red and blue rays is incapable of producing the tendency to turn, while the remaining portion occasions this effect.

INFLUENCE OF ASPHYXIA ON THE SECRETION OF BILE.

The experiments of M. Bouisson on this subject seem to show that *slow* asphyxia causes venous congestion in the liver, and increases considerably the secretion of bile; that bile is produced from the venous blood of the liver; that the qualities of the bile are influenced by slow asphyxia, this fluid becoming more dark and consistent, and appearing to be mixed with blood; finally, that asphyxia, by arresting the functions of the lung, developes the supplementary function of the liver, which throws off the excess of carbon by means of the bile.

SULPHATE OF QUININE IN RHEUMATISM.

M. Gueneau de Mussy read a report on the different papers which had been forwarded to the Academy, on the treatment of acute rheumatism by sulphate of quinine in high doses. After a careful consideration of all the points connected with this disputed question, the committee conclude that the sulphate of quinine should not be prescribed in the high doses of four to six scruples, recommended by M. Briquet; and, secondly, that the same therapeutic effects may be obtained by the ordinary doses of this remedy.—*Ibid.*

